# Insecticidal Activity of Some Traditionally Used Ethiopian Medicinal Plants against Sheep Ked *Melophagus ovinus*

**DOI:** 10.1155/2014/978537

**Published:** 2014-02-04

**Authors:** Negero Gemeda, Walelegn Mokonnen, Hirut Lemma, Ashenif Tadele, Kelbessa Urga, Getachew Addis, Asfaw Debella, Mesaye Getachew, Frehiwot Teka, Kidist Yirsaw, Kissi Mudie, Solomon Gebre

**Affiliations:** ^1^Traditional and Modern Medicine Research Directorate, Ethiopian Health and Nutrition Research Institute, P.O. Box 1242, Addis Ababa, Ethiopia; ^2^National Animal Health Diagnostic and Investigation Center, P.O. Box 04, Sebeta, Ethiopia

## Abstract

Twelve medicinal plants and a commercially used drug Ivermectin were examined for insecticidal activity against *Melophagus ovinus* sheep ked at different time intervals using *in vitro* adult immersion test. The findings show that at 3.13 *µ*L/mL, 6.25 *µ*L/mL and 12.5 *µ*L/mL concentration of *Cymbopogon citratus*, *Foeniculum vulgare *and *Eucalyptus globulus *essential oils respectively, recorded 100% mortalities against *M. ovinus *within 3 hour of exposure. Significantly higher insecticidal activity of essential oils was recorded (*P* = 0.00) when compared to 10 **μ**g/mL Ivermectin after 3-hour exposure of *M. ovinus* at a concentration of ≥1.57 **μ**L/mL, ≥3 **μ**L/mL, and ≥12.7 **μ**L/mL essential oils of *C. citratus*, *F. vulgare*, and *E. globulus*, respectively. Among essential oils, *C. citratus* has showed superior potency at a three-hour exposure of the parasite (*P* = 0.00) at a concentration of ≥0.78 **μ**L/mL. Strong antiparasitic activity was recorded by aqueous extract of *Calpurnia aurea* (80% mortality) at a concentration of 200 mg/mL within 24 h among aqueous extracts of 9 medicinal plants. The results indicated all the four medicinal plants, particularly those tested essential oils, can be considered as potential candidates for biocontrol of *M. ovinus* sheep ked.

## 1. Introduction

In Ethiopia, a livestock sector contribute for 12% of the total Gross Domestic Product (GDP), and provides livelihood for 65% of the population and also it accounts for 37–87% out of the total house hold cash income. For many years not only the export of livestock but also the export of processed and semiprocessed skins and hide constitutes Ethiopia's second largest export commodity by accounting for 12–15% of the total export earnings next to coffee [1, 2]. This has been deteriorating with an increasing number of reject grades and appearance of a skin disease called “Ekek” that is mainly due to ectoparasites infestation [3, 4]. Thirty years ago, tanneries in Ethiopia used to produce 70% of processed skins with grades 1–3. About 10–20% of the skins were graded as being of poor quality. Currently, only 10–15% is in the good category while the rest are downgraded or rejected due to increasing external parasite infestations [5]. Ectoparasite infections have been responsible for major economic loss in leather industry in Ethiopia in which approximately more than half of the skin reject occurs [3, 4, 6].

Among ectoparasites, *Melophagus ovinus* sheep keds are the most economically important pests of sheep [4, 7–9]. *Melophagus ovinus* sheep ked is a voracious blood sucker causing great irritation to the sheep and forcing them to scratch and bite at themselves [10]. The parasites are reported to cause discomfort and annoyance that leads to reduction of weight gain, wool growth, and milk production due to nervousness and improper nutrition of the animals in which they spend less time feeding. Moreover, sheep ked is reported to cause inflammation, and as the host attempts to alleviate this irritation, wool loss and skin damage can be caused. Other report, showed that heavily infested animals become weak and unthrifty and show weight loss, anemia, wool staining, reduced resistance to disease, and a condition called “cockle” which is a series of pimple-like bumps on the skin of the sheep in areas where keds have fed [3, 7]. They reduce the value of the skin. If skins are used in the tanning industry, the bumps will not accept dye like the rest of the pelt and the pelt no longer demands high prices.

In addition, *M. ovinus* sheep ked reduces the growth rates and also causes paralysis and injuries which lead to secondary infection and expose the animal to cutaneous myiasis [11]. Further economic losses result from the effects of feeding and scratching on the skin with hard nodules (cockle) reducing the value of the hide. Wool loss from scratching and biting and the staining of the fleece caused by ked feces in the fleece are further economic effects [10]. Sheep ked also creates financial burdens of diagnostic, therapeutic or preventive programs at flock, community and national levels.

As a result control strategies are looked for to reduce disease and limit losses in the animal husbandry industry to or below acceptable economic damage thresholds [12, 13]. Highly potent chemical insecticides such as organophosphate compounds are routinely and extensively used interventions in ectoparasite control of livestock [5]. However, widespread intensive use of synthetic insecticide has led to pesticide resistance in parasite populations [7, 14, 15]. Besides, their continuous application was coupled with environmental pollution and health concerns by causing the contamination of ground water by particle leachates and toxicity of the chemicals on animals and increasing incidence of human skin, lung, nerve disease upon exposure to these chemicals [7].

Most of the pesticides that are currently used against the various animal ectoparasites in Ethiopia are organophosphorus compounds which cause both contact and stomach poison [16]. They are acting by combining with, and neutralizing cholinesterase action, an enzyme responsible for the hydrolysis of acetylcholine which is synthesized at nerve endings and is involved in the transmission of impulses from nerve to nerve or effecter cells [16, 17]. Previous investigations in Ethiopia have clearly indicated depression of cholinesterase activity associated with symptoms of intoxication among agricultural workers due to lower plasma cholinesterase and erythrocyte cholinesterase of the workers [18, 19].

As the traditional approach to control *M. ovinus* infestations has been only partially successful due to the costs of insecticides, resistance, and environmental contamination, alternative anti-sheep ked products and or control strategies that are safe, cost-effective, and environmentally friendly are therefore necessary [20]. Ethnoveterinary medicine (EVM) specially the use of medicinal plants could be a promising area of alternative sheep ked control strategies. The value of medicinal plants as commercial sheep ked control agents has yet to be proven, but their high safety and effectiveness make them promising candidates for use as commercial sheep ked biocontrol agents [21].

In Ethiopia, there are a number of medicinal plants utilized by EVM practitioners to control ectoparasites infection in livestock. Among them *Aloe* sp. (Family Asparagaceae), *Bersama abyssinica* Fresen. (Melianthaceae), *Calpurnia aurea* (Aition) Benth. (Fabaceae)*, Croton macrostachyus* Hochst. ex Delile. (Euphorbiaceae), *Cymbopogon citratus* (DC.) Stapf (Poaceae)*, Eucalyptus globulus* Labill. (Myrtaceae), *Foeniculum vulgare* Mill. (Apiaceae)*, Jatropha curcas* L. (Euphorbiaceae)*, Nicotiana tabacum* L. (Solanaceae)*, Syzygium guineense *(Willd.) DC. (Myrtaceae)*, Vernonia auriculifera* Hiern. (Asteraceae), and *Ximenia caffra *Sond. (Olacaceae) were mainly used in EVM [22–28]. The study was therefore conducted to evaluate the *in vitro* bioinsecticidal efficacy of indigenous plants used in EVM practice in Ethiopia for the control of *M. ovinus* sheep ked.

## 2. Material and Methods

### 2.1. Experimental Parasite Collection and Maintenance

*Melophagus ovinus* sheep ked parasite was used in this study to conduct *in vitro* insecticidal assay of medicinal plants. Sheep keds were collected from naturally infested sheep around Fiche, North Shewa, Oromia Zone, Ethiopia. The identity of the parasites was confirmed on site by veterinary professionals found in the collection area. After their identification, the parasites were maintained in plastic cups into which water soaked cottons were placed to increase the humidity of the air found in the cups. The cups were covered by gauze to allow the free circulation of air into the cups. And the parasites were transported to the Fiche Animal Health Clinic Laboratory where the experimental works were conducted.

### 2.2. Plant Material Collection, Identification, and Extraction

*Eucalyptus globulus* and *Cymbopogon citratus* essential oils were obtained from Wondo Genet Agricultural Research Center, Ethiopia. Essential oils of *Foeniculum vulgare* Mill. were extracted by hydrodistillation of the fresh leaf which was collected from Shashamane, Ethiopia. Fresh plant materials (250 g) were placed in a 5 L round-bottom distillation flask and the plant material was wetted with 3 L distilled water. The essential oils were obtained by hydrodistillation using Clevenger-type apparatus for continuous 3 h. The volatile oil was taken from the upper layer. The excess aqueous layers were further portioned using dichloromethane extract and enrich the essential oil from the water layer. The organic layer (dichloromethane extract) was filtered and dried with anhydrous sodium sulfate and concentrated using rotary evaporator to give the crude essential oil.

Three hundred grams of dry powdered leaves of *Calpurnia aurea* (leaves)*, Syzygium guineense *(leaves)*, Bersama abyssinica* (leaves)*, Croton macrostachyus* (leaves), *Nicotiana tabacum *(leaves)*, Vernonia auriculifera* (leaves), *Aloe species* (aerial part), and the fruits of *Jatropha curcas* and *Ximenia caffra* was macerated with 25–50 mL water. The aqueous extracts were filtered using gauze and freeze dried using lyophilizer. The volatile oils and the amorphous residues from the aqueous macerate are kept in the refrigerator and desiccators, respectively, until used for the *in vitro* insecticidal susceptibility assay.

### 2.3. Insecticidal Assay

#### 2.3.1. Adult Immersion Test

Insecticidal activity of 12 plant extracts was performed using adult immersion test according to Drummond et al. (1976) as cited in [29]. Twofold serial dilutions of plants essential oils (50 *μ*L/mL, 25 *μ*L/mL, 12.5 *μ*L/mL, 6.25 *μ*L/mL, 3.125 *μ*L/mL, 1.5625 *μ*L/mL, and 0.78125 *μ*L/mL) were prepared in 2% aqueous solution of Tween 80. Similarly, twofold serial dilutions of aqueous plants extracts (200 mg/mL, 100 mg/mL, 50 mg/mL, 25 mg/mL, 12.5 mg/mL, and 6.25 mg/mL) were prepared in distilled water. Antiparasitic effects of each dilution were tested by immersing a group of 10 *M. ovinus* parasites in a Petri dish containing 3–5 mL of the extracts for 1 minute. Two percent Tween 80 and water were used as negative control and 10 *μ*g/mL Ivermectin was used as positive control. The experiment was performed in triplicate and the Petri dishes were incubated at 27-28°C and 80% relative humidity for 24 h. The parasites were studied with stereomicroscope and the mortality was recorded at 3 h interval till 24 h by counting dead parasites (alive and dead) and the corrected mortality rate of 3 h and 24 h was reported. The percent mortality rate of the tick was calculated as per Abotts (1925) as cited in [30]:

(1) Corrected  Mortality =%Treated  mortality−%control  mortality100−%control  mortality×100.

### 2.4. Data Analysis

Insecticidal effect was classified as follows: strong, mortality >80%; moderate, mortality 80–60%; weak, mortality 60–40%; little or no activity, mortality < 40%. Mortality in the Petri dishes treated with extract was corrected to take account of control mortality using Abbott's correction. The mortality rates were determined and transformed and subjected to analysis of variance (ANOVA) using statistical software (Minitab 16.0, England). Post hoc testing was done using Tukey. Percent mortality and values were presented as mean ± S.D. Insecticidal activity was considered to be significantly different when 95% confidence limit levels failed to overlap or if *P* value < 0.05.

## 3. Results

A total of 12 extracts from 12 different medicinal plants were tested for insecticidal activity against *M. ovinus*. The insecticidal activity of essential oils of *C. citratus, E. globulus,* and *F. vulgare* at a concentration of 0.7825, 1.565, 3.13, 6.25, 12.5, 25, and 50 *μ*L/mL against adult *M. ovinus* sheep ked is shown in Table 1. The result showed that *C. citratus* essential oil has pronounced insecticidal activity followed by *F. vulgare* and *E. globulus*. Hundred percent mortalities were recorded by *C. citratus, F. vulgare,* and *E. globulus* against *M. ovinus* within 3 h of exposure at a concentration of 3.13 *μ*L/mL, 6.25 *μ*L/mL, and 12.5 *μ*L/mL, respectively. Sheep ked mortality increased as concentration and exposure time to the essential oil increased.

Statistical results (one-way ANOVA (unstacked)) have showed significantly higher insecticidal activity of essential oils (*P* < 0.01) after a 3 h exposure of *M. ovinus* to concentrations of ≥1.57 *μ*L/mL, ≥3 *μ*L/mL, and ≥12.7 *μ*L/mL essential oils of *C. citratus, F. vulgare,* and *E. globulus,* respectively, as compared with 10 *μ*g/mL Ivermectin. Among the essential oils, at 3 h exposure of the parasite, significantly higher (*P* < 0.01) insecticidal activity was exerted by *C. citratus* at a concentration ≥0.78 *μ*L/mL.

Mortality of *M. ovinus* sheep keds exposed to aqueous extract of *C. aurea*, *X. caffras* and *J. curcas* at concentration of 6.25, 12.5, 25, 50, 100, and 200 mg/mL is shown in Table 2. The maximum antiparasitic activity was exerted by aqueous extract of *C. aurea*. Eighty percent, 60% and 66.3% mortality of *M. ovinus* sheep keds were observed after 24 h of exposure to 200 mg/mL of *C. aurea, X. caffra,* and *J. curcas,* respectively. The highest insecticidal efficacy was recorded by *C. aurea* with (73.3%) mortality followed by *X. caffra* (60%) and *J. curcas *(56.7%) at a concentration of 200 mg/mL, within 3 hours of the parasite exposure.

Table 3 indicated the antiparasitic activity of different concentrations of aqueous extracts of *S. guineense, N. tabacum,* and *V. auriculifera* against *M. ovinus* sheep keds. A 43.5% mortality of sheep keds was recorded by *S. guineense* at a concentration of 200 mg/mL within 3 h of exposure, while a comparatively lower mortality rate (20%) was recorded by both *N. tabacum* and *V. auriculifera* within 3 h of exposure. All extracts exerted significantly lower insecticidal effect when compared to 10 *μ*g/mL Ivermectin (*P* < 0.05).

The effects of Aloe sp., *B. abyssinica,* and *C. macrostachyus* on *M. ovinus* sheep ked are also shown in Table 4. The exposure of *M. ovinus* sheep ked to the dose of 200 mg/mL of *Aloe* sp*.,* were showed mortality of 30% within 3 h. Relatively, a higher antiparasitic potential was recorded by *Aloe* sp. than the other species.

## 4. Discussion

Though Ethiopia has a potential of supplying livestock and livestock products, the sector is facing multiple challenges and ectoparasites continued to be highly prevalent; even the most commonly used insecticides are becoming ineffective due to the development of resistance in addition, environment pollution and health risk of the insecticide [7]. These problems had stimulated a great deal of research to look for alternative treatment from natural products. For this purpose we evaluated activity of twelve traditionally used Ethiopian medicinal plants for the alternative management of sheep ked infestation. Application of plants essential oils or plant extracts for the control of pest has been used for hundreds of years by the community and practitioners of traditional medicine. Similarly, various studies showed the antiparasitic and insecticidal properties of the plant essential oils against phytophagous pests, ticks [31], and mites [22, 23].

Our findings have confirmed that essential oils from *C. citratus, E. globulus,* and *F. vulgare *have antiparasitic activity against *M. ovinus*. Essential oil of *C. citratus* has pronounced insecticidal activity followed by *F. vulgare* and *E. globulus*. Complete immobility of the sheep ked parasite was recorded at a concentration of 3.13 *μ*L/mL, 6.25 *μ*L/mL, and 12.5 *μ*L/mL essential oils of *C. citrates, F. vulgare,* and *E. globules,* respectively. The insecticidal effect of the essential oils are depends on both dose and type of plant species as the significant effect was recorded at higher dose level. Moreover, the highest mortality effect against *M. ovinus* was observed by *C. citratus* which is considered as the best insecticidal agent among the investigated plants. Our finding that essential oils have insecticidal activity is in line with several studies where they indicated that many essential oils could possibly substitute synthetic chemicals [32], with higher insecticidal effect of *C. citratus* against mite [26], insecticidal potentials of *F. vulgare* fruit against mite and a significant dose dependent insecticidal effect of *E. globulus* against tick [33]. The mortality of the parasite may be due to the octopaminergic nervous system interference by these essential oils [34]. Similarly, the activity could be due to complex mixture of secondary metabolites like terpenoids, phenolics of mono-, di- and sesquiterpenes, specifically citral, elemicin, (+)-fenchone, and p-anisaldehyde, and 1,8-cineole in the essential oils [34]. The difference in bioactivity between the essential oils may be due to the difference in major compounds, as biological activity of essential oils depends on chemical structure of their components [35, 36]. Moreover, chemical components may be toxic to various pests individually or in combination. Activity may be also influenced by the arrangement of multiple components in the essential oils as a result of synergistic effects [23].

Another notable finding of the current study was that aqueous extract of plants has insecticidal activity. Promising activity (80% mortality) was recorded by *C. aurea*, while 60% mortality by *X. caffra,* and about 57% mortality by *J. curcas* are still worth a credit as potential source of botanical insecticide. Our study that confirmed *C. aurea* has remarkable antiparasitic activity replicates a previous study [24], which reported 85% mortality of tick at 5% concentration of *C. aurea*. Moreover, aqueous extracts of *J. curcas* and *X. caffra *have a potential of killing or immobilizing *M. ovinus* sheep keds which is in line with previous study [27].

Moreover, the finding of current study showed that aqueous extracts of *S. guineense, N. tabacum,* and *V. auriculifera* have moderate toxicity against *M. ovinus* sheep keds. A mortality of 47% was recorded within 24 h of exposure of sheep ked parasite to 200 mg/mL of *S. guineense*. The antiparasitic effect observed by *S. guineense* was also supported by the findings of closely related species [25] on the ethanolic extract of *Syzygium cumini* that showed the highest antiparasitic (98.5% mortality) effect. Our findings also replicate the finding of Zaman et al. [28] whereby mortality of tick parasite was reported to be in combination with application of *Azadirachta indica* and *N. tabacum* leaves, *Calotropis procera* flowers, and *Trachyspermum ammi* seeds aqueous extracts.

Many of the plants used in this study which showed insecticidal activity are reported to have ethnoveterinary medicine practices in Ethiopia as elsewhere in the world [37]. However, our finding showed that aqueous extracts of *Aloe* sp.*, B. abyssinica,* and *C. macrostachyus* have lower lethality activity against *M. ovinus* when compared to other plants and that of the commercially used antiparasite Ivermectin. This could be due to the extraction method we used; one can find higher activity by using other extractants than water. Similarly, various studies reported the antihelminthic activity of *Aloe* sp. [38] and the antimicrobial activity of *B. abyssinica* [39] and *C. macrostachyus* [40]. This could be an indication of the broad spectrum activity of the plant extracts.

## 5. Conclusion

The current study showed that the essential oils of *C. citratus, E. globulus,* and *F. vulgare* and the aqueous extract of *C. aurea* have strong insecticidal activity. The results of this study offer some scientific credence to the traditional uses of the Ethiopian medicinal plants evaluated for the management of Sheep ked parasites. There are many opportunities for the use of plant essential oils as alterative to synthetic insecticide; one is their insecticidal potency and environmental sound nature, easy accessibility by the rural pastoralist community, and superiority over synthetic antiparasite Ivermectin. Another important opportunity is their easy application as fumigant and topical treatment. Regarding *C. aurea*, it can be one potential alternative to synthetic insecticide to control sheep ked which is advantageous by its wide occurrence in Ethiopia and its capacity to resist drought and overgrazing. There is, however, a need for further investigation on their safety and efficacy (*in vivo* and under natural conditions) as well as cost-effectiveness of the products that exhibited strong insecticidal activity with a view of substituting the conventional organophosphorus drugs. And also we recommend bioassay guided fractionation, isolation, and characterization of the responsible active components of the plant materials.

## Figures and Tables

**Table 1:** The corrected mortalities of *M. ovinus* sheep keds exposed to different concentrations of *C. citratus*, *F. vulgare*, and *E. globulus* essential oils from the leaves.

Dose (mg/mL)	Corrected mortality rate (%)					
	*E. globulus *		*C. citratus *		*F. vulgare *	
	3 h PI	24 h PI	3 h PI	24 h PI	3 h PI	24 h PI
0.7825	0.00 ± 0.0^d^	0.00 ± 0.0^c^	16.7 ± 0.6^c^	43.3 ± 1.15^b^	0.00 ± 0.0^e^	0.00 ± 0.0^d^
1.565	0.00 ± 0.0^d^	6.70 ± 0.6^c^	93.3 ± 0.6^a^	93.3 ± 0.6^a^	53.3 ± 0.6^d^	53.3 ± 1.2^c^
3.13	56.7 ± 0.6^c^	66.7 ± 1.2^b^	100.0 ± 0.0^a^	100.0 ± 0.0^a^	86.7 ± 0.6^b^	86.7 ± 0.6^b^
6.25	83.3 ± 1.0^b^	86.7 ± 0.6^a^	100.0 ± 0.0^a^	100.0 ± 0.0^a^	100.0 ± 0.0^a^	100.0 ± 0.0^a^
12.5	100.0 ± 0.0^a^	100.0 ± 0.0^a^	100.0 ± 0.0^a^	100.0 ± 0.0^a^	100.0 ± 0.0^a^	100.0 ± 0.0^a^
25	100.0 ± 0.0^a^	100.0 ± 0.0^a^	100.0 ± 0.0^a^	100.0 ± 0.0^a^	100.0 ± 0.0^a^	100.0 ± 0.0^a^
50	100.0 ± 0.0^a^	100.0 ± 0.0^a^	100.0 ± 0.0^a^	100.0 ± 0.0^a^	100.0 ± 0.0^a^	100.0 ± 0.0^a^
Control^+^	73.3 ± 0.6^b^	100.0 ± 0.0^a^	73.3 ± 0.6^b^	100.0 ± 0.0^a^	73.3 ± 0.6^c^	100.0 ± 0.0^a^

^+^Ivermectin (10 *μ*g/mL); PI: postincubation; values are expressed as mean ± SD. Mean values with different letters in the same column are significantly different (*P* < 0.05).

**Table 2:** The corrected mortalities of *M. ovinus* sheep keds exposed to different concentrations of aqueous extracts of *C. aurea* leaves, *X. caffra* fruits, and *J. curcas* fruits.

Dose (mg/mL)	Corrected mortality rate (%)					
	*C. aurea *		*X. caffra *		*J. curcas *	
	3 h PI	24 h PI	3 h PI	24 h PI	3 h PI	24 h PI
6.24	0.0 ± 0.0^d^	0.0 ± 0.0^a^	0.0 ± 0.0^d^	0.0 ± 0.0^d^	0.0 ± 0.0^c^	0.0 ± 0.0^e^
12.5	13.3 ± 0.6^cd^	16.7 ± 0.6^d^	10.0 ± 0.0^cd^	10.0 ± 0.0^d^	0.0 ± 0.0^c^	6.7 ± 0.6^e^
25	13.3 ± 0.6^cd^	23.3 ± 0.6^d^	20.0 ± 1.0^bc^	23.3 ± 0.6^c^	6.7 ± 0.6^bc^	13.3 ± 0.6^de^
50	26.7 ± 0.6^c^	27.8 ± 1.0^d^	26.7 ± 0.6^b^	30.0 ± 0.0^c^	16.7 ± 1.0^bc^	23.3 ± 0.6^cd^
100	53.3 ± 0.6^b^	63.3 ± 0.6^c^	33.3 ± 0.6^b^	50.0 ± 1.0^b^	23.3 ± 1.0^b^	33.3 ± 0.6^c^
200	73.3 ± 0.6^a^	80.0 ± 0.0^b^	60.0 ± 0.0^a^	60.0 ± 0.0^b^	56.7 ± 0.6^a^	66.3 ± 0.6^b^
Control^+^	73.3 ± 0.6^a^	100.0 ± 0.0^a^	73.3 ± 0.6^a^	100.0 ± 0.0^a^	73.3 ± 0.6^a^	100.0 ± 0.0^a^

^+^Ivermectin (10 *μ*g/mL); PI: postincubation; values are expressed as mean ± SD. Mean values with different letters in the same column are significantly different (*P* < 0.05).

**Table 3:** Percent corrected mortality of *M. ovinus* sheep keds exposed to different concentrations of *S. guineense*,  *N. tabacum*, and *V. auriculifera* aqueous extracts.

Dose (mg/mL)	Corrected mortality rate (%)					
	*S. guineense *		*N. tabacum *		*V. auriculifera *	
	3 h PI	24 h PI	3 h PI	24 h PI	3 h PI	24 h PI
6.24	0.0 ± 0.0^e^	0.0 ± 0.0^d^	0.0 ± 0.0^c^	0.0 ± 0.0^e^	0.0 ± 0.0^c^	0.0 ± 0.0^e^
12.5	3.3 ± 0.6^de^	6.7 ± 0.6^d^	0.0 ± 0.0^c^	6.6 ± 0.6^de^	6.7 ± 0.6^bc^	6.7 ± 0.6^de^
25	13.3 ± 0.6^cd^	13.9 ± 0.6^cd^	6.6 ± 0.6^c^	16.7 ± 0.6^cd^	10.0 ± 0.0^bc^	13.3 ± 0.6^cde^
50	20.0 ± 0.0^c^	23.3 ± 0.6^c^	10.0 ± 0.0^c^	20.0 ± 0.0^c^	10.0 ± 0.0^bc^	16.7 ± 0.6^cd^
100	40.0 ± 0.0^b^	43.3 ± 0.6^b^	13.3 ± 0.6^c^	36.7 ± 0.6^b^	16.7 ± 0.6^b^	23.3 ± 0.6^c^
200	43.5 ± 0.6^b^	46.7 ± 0.6^b^	20.0 ± 0.0^b^	43.3 ± 0.6^b^	20.0 ± 0.0^b^	43.3 ± 0.6^b^
Control^+^	73.3 ± 0.6^a^	100.0 ± 0.0^a^	73.3 ± 0.6^a^	100.0 ± 0.0^a^	73.3 ± 0.6^a^	100.0 ± 0.0^a^

^+^Ivermectin (10 *μ*g/mL); PI: postincubation; values are expressed as mean ± SD. Mean values with different letters in the same column are significantly different (*P* < 0.05).

**Table 4:** The corrected mortality rate of *M. ovinus* sheep keds exposed to different concentrations of *Aloe *species,  *B. abyssinica*, and *C. macrostachyus* leaves aqueous extracts.

Dose (mg/mL)	Corrected mortality rate (%)					
	*Aloe *sp.		*C. macrostachyus *		*B. abyssinica *	
	3 h PI	24 h PI	3 h PI	24 h PI	3 h PI	24 h PI
6.24	0.0 ± 0.0^d^	0.0 ± 0.0^c^	0.0 ± 0.0^d^	0.0 ± 0.0^d^	0.0 ± 0.0^d^	0.0 ± 0.0^d^
12.5	6.7 ± 0.6^cd^	6.7 ± 0.6^c^	6.7 ± 0.6^d^	6.7 ± 0.6^d^	0.0 ± 0.0^d^	0.0 ± 0.0^d^
25	13.3 ± 0.6^c^	13.3 ± 0.6^c^	6.7 ± 0.6^d^	6.7 ± 0.6^cd^	0.0 ± 0.0^d^	6.7 ± 0.6^cd^
50	13.3 ± 0.6^c^	13.3 ± 0.6^c^	6.7 ± 0.6^cd^	6.7 ± 0.6^bc^	6.7 ± 0.6^cd^	16.7 ± 0.6^bc^
100	30.0 ± 0.0^b^	40.0 ± 1.0^b^	26.7 ± 0.6^bc^	26.7 ± 0.6^b^	16.7 ± 0.6^bc^	20.0 ± 0.0^b^
200	30.0 ± 0.0^b^	40.0 ± 1.0^b^	30.0 ± 0.0^b^	30.0 ± 0.0^b^	23.3 ± 0.6^b^	26.7 ± 0.6^b^
Control^+^	73.3 ± 0.6^a^	100.0 ± 0.0^a^	73.3 ± 0.6^a^	100.0 ± 0.0^a^	73.3 ± 0.6^a^	100.0 ± 0.0^a^

^+^Ivermectin (10 *μ*g/mL); PI: postincubation; values are expressed as mean ± SD. Mean values with different letters in the same column are significantly different (*P* < 0.05).
